# Case report: Stercoral sigmoid colonic perforation with fecal peritonitis

**DOI:** 10.4103/0971-3026.63051

**Published:** 2010-05

**Authors:** Monika Sharma, Anjali Agrawal

**Affiliations:** Department of Radiology, Teleradiology Solutions, 12B, Shri Ram Road, Civil Lines, New Delhi-110052, India

**Keywords:** Constipation, fecal impaction, peritonitis, stercoral perforation

## Abstract

Chronic constipation can lead to fecal impaction. It can also rarely lead to catastrophic complications like perforation, colonic obstruction, and fecal peritonitis. We report a rare case of stercoral sigmoid colonic perforation with fecal peritonitis and pneumoperitoneum, which was diagnosed on preoperative CT scan.

## Introduction

Stercoral peritonitis is fecal contamination of the peritoneal cavity due to various causes, most commonly diverticular disease and colorectal tumor. Stercoral peritonitis is associated with a high morbidity and mortality rate; therefore an early diagnosis is important.[1] Stercoral colitis is an inflammatory colitis related to fecal impaction, which results in ischemic pressure necrosis of the rectal and colonic walls, usually the sigmoid colon, due to increased intraluminal pressure; this leads to stercoral ulcer formation and, subsequently, colonic perforation.[2] The aim of this case report is to discuss the CT scan findings of stercoral colonic perforation. A correct preoperative diagnosis can facilitate early surgical intervention and thus prevent or mitigate serious complications.

## Case Report

**Figure 1 (A, B):**
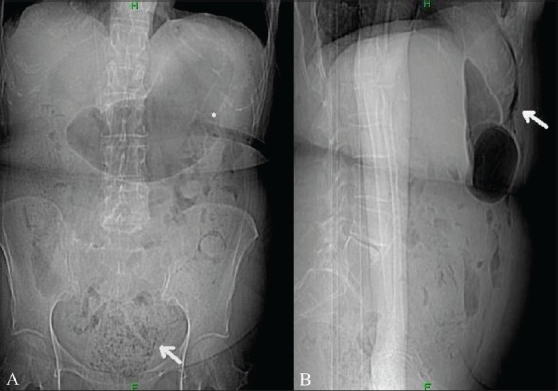
Anteroposterior (A) and lateral (B) scout CT scan images show abundant fecal material and fecalomas in the pelvis (arrow in A) and left colon and pneumoperitoneum (arrow in B)

**Figure 2:**
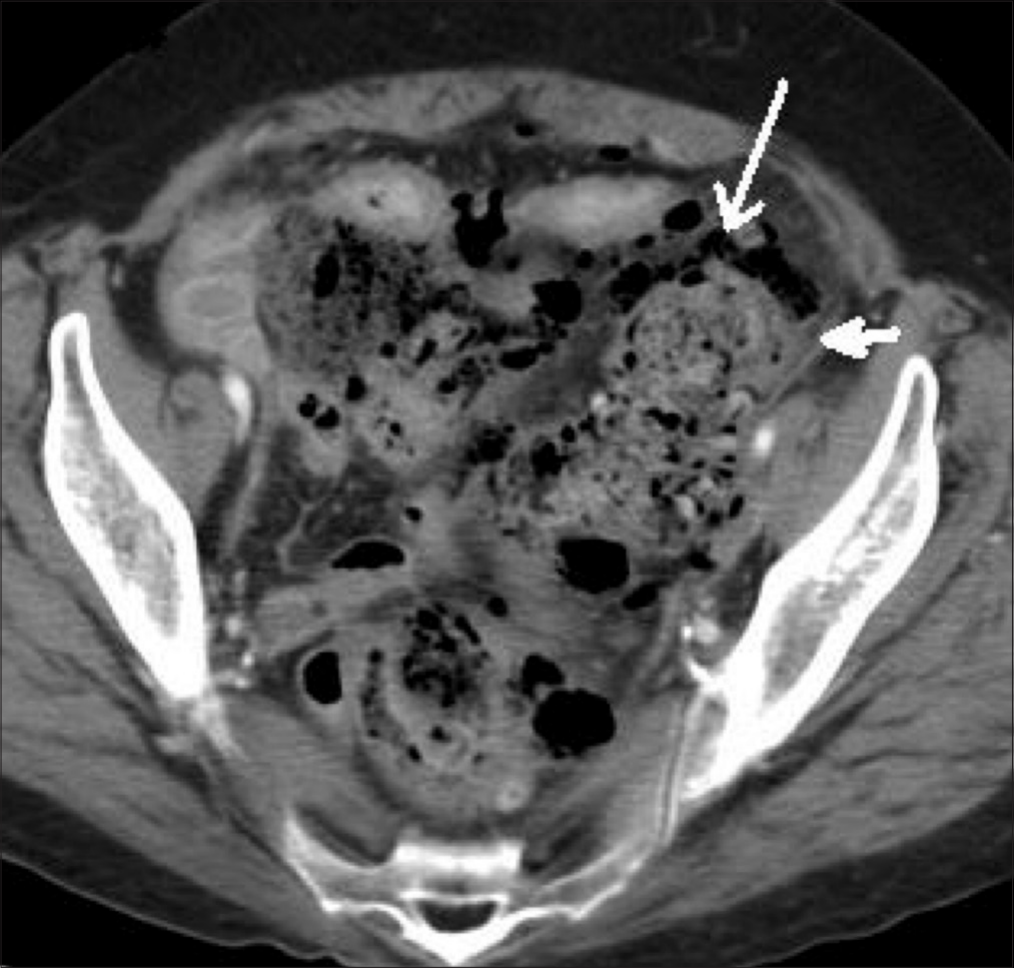
Contrast-enhanced axial CT scan demonstrates a fecaloma (arrow) in the proximal sigmoid colon and at the site of the perforation, with colonic wall thickening (arrowhead) due to pressure necrosis

**Figure 3:**
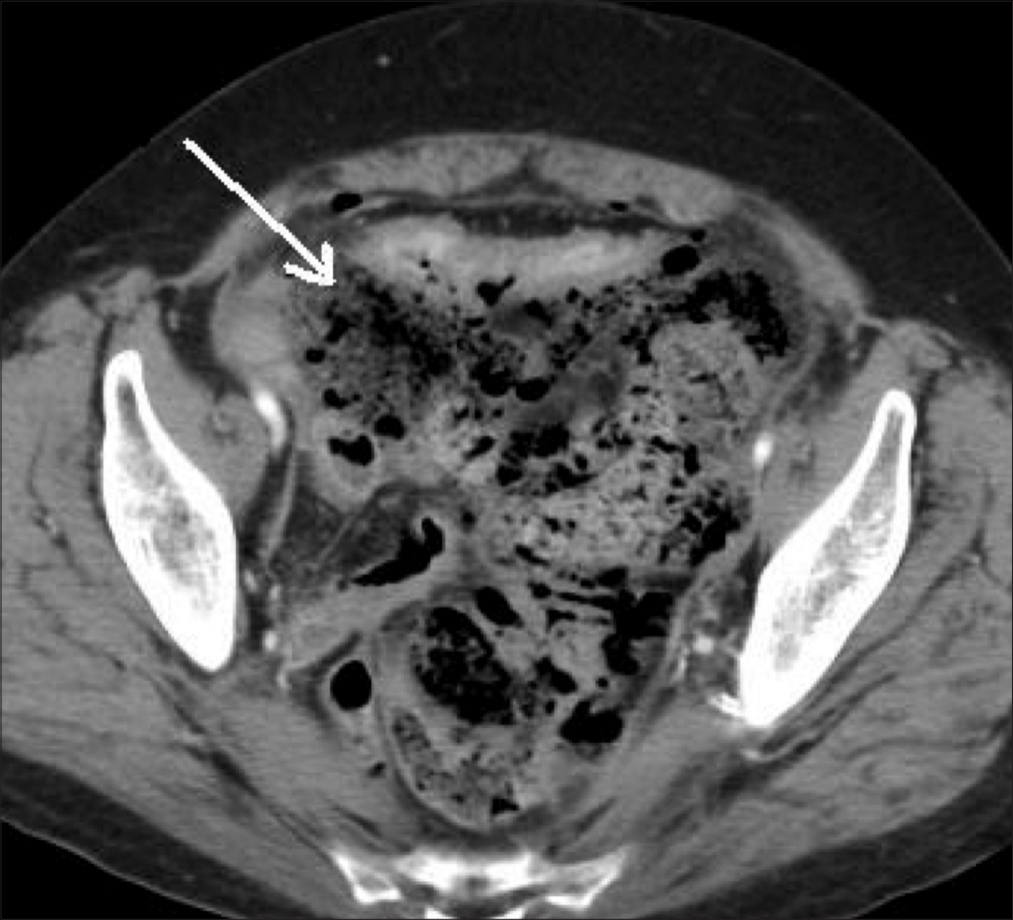
Contrast-enhanced axial CT scan shows extensive extraluminal fecal matter in the peritoneal cavity (arrow) and air loculi, consistent with fecal peritonitis

A 67-year-old female was admitted in the emergency department with a history of constipation for 5 days, abdominal pain, and increasing abdominal distension. Her WBC count was within normal limits (WBC count may not be elevated in elderly patients presenting with an acute abdomen). On physical examination, the patient had a distended abdomen and signs of peritonitis. CT scan of abdomen and pelvis was performed with oral and intravenous contrast. Scout antero-posterior (AP) and lateral images demonstrated free air and abundant fecal material in the pelvis [Figures 1A, B]. Contrast-enhanced axial CT scan images demonstrated abundant fecal matter in the rectum and sigmoid colon, which were dilated. There was extensive extraluminal fecal matter in the pelvis, focal thickening of the sigmoid colon, and moderate pneumoperitoneum [Figures 2 and 3]. Based on these CT scan findings, the diagnosis of stercoral perforation with fecal peritonitis was made. Surgical exploratory laparotomy of the abdomen was performed, which confirmed the CT scan diagnosis. Peroperatively, the rectosigmoid colon was distended with abundant fecal material and a focal defect was seen in the proximal sigmoid colon. The pelvic intraperitoneal cavity was extensively contaminated with fecal matter. Peritoneal irrigation was done, with resection of the sigmoid colon and end-colostomy. The patient's postoperative course was unremarkable.

## Discussion

Stercoral perforation is a rare surgical condition, with fewer than 90 cases reported in the literature upto 2002.[3,4] This condition was first described by Berry in 1894[4] and is defined as colonic perforation due to the pressure effect of hard fecalomas on the wall of an otherwise normal colon, in the absence of any other established pathology.[5]

The pathogenesis is related to poor hydration of the feces, resulting in hard and impacted feces, also called fecaloma. This increases intraluminal pressure and causes ischemic necrosis of the colonic wall, stercoral ulcer formation and, subsequently, colonic perforation.[2] The most common locations (more than 90% of the times) for stercoral ulceration and perforation are the anterior rectum (just proximal to the peritoneal reflection), the antimesenteric border of the rectosigmoid junction, and the sigmoid colon.[2,3] related to decreased perfusion at the antimesenteric border of the sigmoid and rectosigmoid colon.[4,2] as well the narrow diameter of the distal colon, due to which it is difficult for hard stools to pass through the sigmoid colon, leading to increased intraluminal pressure and colonic ischemia.[4]

The mean age of presentation in such cases is 59 years and the age range is 22–85 years.[6] In 60% of cases of fecal impaction, there is a prior history of constipation.[6] There are a few risk factors for stercoral ulcer perforation, which include chronic intermittent constipation and the use of nonsteroidal anti-inflammatory drugs or drugs-like amitryptyline, antacids, steroids, codeine, and heroin.[3,7,8] On CT scan, the diagnosis of fecal impaction is obvious, with visualization of a dilated rectum and colon containing dense, lamellated fecal masses (fecalomas) with thin colonic walls. The CT scan findings of stercoral colitis include focal colonic or rectal wall thickening involving the dilated sigmoid colon and rectum, which demonstrate fecalomas. Pericolonic/perirectal fat stranding is usually seen due to colonic ischemia or wall edema.[2] The presence of intramural or extraluminal air loculi suggests colonic perforation. Maurer *et al*.[6] described a few diagnostic criteria for stercoral perforation; these include (1) a rounded or ovoid colonic perforation, which is more than 1 cm in diameter and antimesenteric in location; (2) the presence of fecalomas within the colon, protruding through the perforation site, or found free within the abdominal cavity; and (3) pressure necrosis, ulcer formation, and chronic inflammatory reaction around the perforation site microscopically. According to these criteria, the presence of any underlying colonic pathology, such as diverticulitis, infectious process, inflammatory bowel disease, or obstruction, excludes the diagnosis of primary stercoral perforation of the colon. It is important to diagnose stercoral colitis preoperatively particularly in elderly patients with severe chronic constipation or long history of use of constipating drugs and less commonly, young patients who are neurologically impaired.[2,8] CT scan is particularly important in the preoperative diagnosis in the elderly age group since the laboratory findings may not always correlate with the actual pathology. If this condition is not treated promptly, it can be fatal, with a 35% mortality rate.[2]

## Conclusion

We conclude that in patients with a history of severe or chronic constipation presenting with acute symptoms, the possibility of stercoral perforation should be considered.

## References

[1] Hoch J (1999). Stercoral Peritonitis.

[2] Heffernan C, Pachter HL, Megibow AJ, Macari M (2005). Stercoral colitis leading to fatal peritonitis: CT findings. AJR Am J Roentgenol.

[3] Patel VG, Kalakuntla V, Fortson JK, Weaver WL, Joel MD, Hammami A (2002). Stercoral perforation of the sigmoid colon: report of a rare case and its possible association with nonsteroidal anti-inflammatory drugs. Am Surg.

[4] Huang WS, Wang CS, Hsieh CC, Lin PY, Chin CC, Wang JY (2006). Management of patients with stercoral perforation of the sigmoid colon: Report of five cases. World J Gastroenterol.

[5] Arvind N, Gowrisankar A, Rajkumar JS (2006). Primary stercoral perforation of the colon- rare, but deadly. Indian J Surg.

[6] Maurer CA, Renzulli P, Mazzucchelli L, Egger B, Seiler CA, Büchler MW (2000). Use of accurate diagnostic criteria may increase incidence of stercoral perforation of the colon. Dis Colon Rectum.

[7] Tessier DJ, Harris E, Collins J, Johnson DJ (2002). Stercoral perforation of the colon in a heroin addict. Int J Colorectal Dis.

[8] Hollingworth J, Alexander-Williams J (1991). Non-steroidal anti-inflammatory drugs and stercoral perforation of the colon. Ann R Coll Surg Engl.

